# *QuickStats:* Percentage* of Children and Teens Aged 5–17 Years Who Missed >10 School Days in the Past 12 Months Because of Illness or Injury,^†^ by Sex and Age — National Health Interview Survey, 2013–2015^§^

**DOI:** 10.15585/mmwr.mm6626a8

**Published:** 2017-07-07

**Figure:**
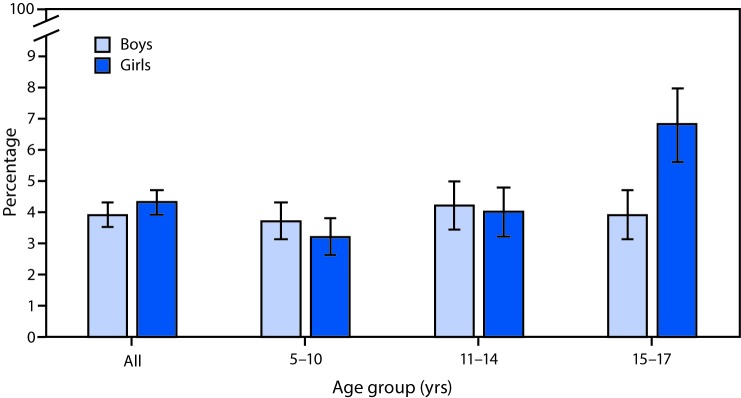
During 2013–2015, 3.9% of boys and 4.3% of girls missed >10 school days in the past 12 months because of illness or injury. Among children aged 15–17 years, girls were more likely than boys to miss >10 school days (6.8% compared with 3.9%). Among girls, those aged 15–17 years were more likely than girls aged 5–10 years and girls aged 11–14 years to miss >10 school days (6.8% compared with 3.2% and 4.0%, respectively). Among boys, there was no difference by age.

**Source:** National Center for Health Statistics. National Health Interview Survey, 2013–2015. https://www.cdc.gov/nchs/nhis.htm.

